# Telemedicine in Cardiology: Enhancing Access to Care and Improving Patient Outcomes

**DOI:** 10.7759/cureus.62852

**Published:** 2024-06-21

**Authors:** Oluwaremilekun Tolu-Akinnawo, Francis Ezekwueme, Toluwalase Awoyemi

**Affiliations:** 1 Internal Medicine, Meharry Medical College, Nashville, USA; 2 Internal Medicine, University of Pittsburgh Medical Center, Pittsburg, USA; 3 Internal Medicine, Nuffield Department of Women's and Reproductive Health, University of Oxford, Oxford, GBR

**Keywords:** preventative cardiology, health-care equity, assess healthcare, telemedicine ai, telemedicine in cardiology

## Abstract

Telemedicine has gained significant recognition, particularly since the COVID-19 pandemic. However, its roots date back to its significant role during major epidemic outbreaks such as severe acute respiratory syndrome (SARS), H1N1 and H7N9 influenza, and Middle East respiratory syndrome (MERS), where alternate means of accessing healthcare were adopted to combat the outbreak while limiting the spread of the virus. In Sub-Saharan Africa, telemedicine has supported healthcare delivery, patient and professional health education, disease prevention, and surveillance, starting with its first adoption in Ethiopia in 1980. In the United States, telemedicine has significantly impacted cardiology, particularly at-home monitoring programs, which have proven highly effective for patients with abnormal heart rhythms. Devices such as Holter monitors, blood pressure monitors, and implantable cardioverter-defibrillators have reduced mortality rates and hospital readmissions while improving healthcare efficiency by saving healthcare costs. However, the COVID-19 pandemic accelerated the adoption of telemedicine, as evidenced by a dramatic increase in telemedicine visits at institutions like New York University (NYU) Langone Health during and post-COVID-19 pandemic. In addition, telemedicine has also facilitated cardiac rehabilitation and improved access to specialized cardiology care in rural and underserved areas, reducing disparities in cardiovascular health outcomes. As technology advances, telemedicine is poised to play an increasingly significant role in cardiology and healthcare at large, enhancing patient management, healthcare efficiency, and cost reduction. This review underscores the significance of telemedicine in cardiology, its challenges, and future directions.

## Introduction and background

The history of telecardiology dates back to the early 1990s when primitive forms of telecommunication were first used to transmit electrocardiograms (ECGs) over telephone lines [1]. Over the last decades, the field has significantly evolved, particularly gaining momentum with advancements in digital technology and the internet. Telecardiology, a subfield of telemedicine, utilizes telecommunication technologies to deliver cardiac care remotely [2]. Although telecardiology was initially adopted to potentially extend to remote and underserved areas to curb health disparities, telemedicine has steadily grown in popularity among healthcare providers and patients. This is driven by the triple imperatives of convenience, improved patient care, and reduced healthcare costs [3].

Today, telecardiology encompasses a wide range of applications in the cardiology world, including remote patient monitoring. This promotes continuous surveillance of patients with chronic heart conditions, enabling early detection of potential issues and timely intervention [4]. Through telecardiology, teleconsultations have facilitated access to specialist advice without needing physical travel, particularly benefiting patients in rural or underserved regions [5] and patients with tight schedules. Additionally, telecardiology supports the remote diagnosis and management of acute cardiac events, such as myocardial infarctions, through real-time data transmission and collaboration between primary care providers and cardiologists [6], thereby further improving patient health outcomes.

Improving technological advancements and expanding applications have marked the evolution of telecardiology. Wearable technology has become particularly advanced, including sophisticated devices capable of continuously monitoring vital signs such as heart rhythm, blood pressure, and oxygen levels. The advent of wearable devices and mobile health applications has empowered patients to participate actively in their care, providing clinicians with real-time health data, including imaging and laboratory results. This has facilitated a more integrated and holistic approach to healthcare, providing a multidisciplinary pathway to the cardiology world where teams can collaborate effectively to provide the best care for patients and improve healthcare efficiency. As the field advances, telecardiology promises to play an increasingly pivotal role in delivering efficient, effective, patient-centered cardiac care.

Additionally, the integration of artificial intelligence (AI) and machine learning into the field of telecardiology poses a promising future due to its ability to analyze real-time data, enhance diagnostic quality, and predict outcomes while still aiding in the detection of cardiovascular conditions such as arrhythmias and heart failure [7]. Its ability to predict outcomes early is critical to early health-seeking behavior and intervention [7]. This, in turn, improves patient outcomes and healthcare efficiency by reducing healthcare costs. However, there are still potential barriers to the widespread adoption of telemedicine, including implementation in rural and low-income areas with limited access to advanced technological infrastructures and high-speed internet, raising the concern for worsening health disparity. Also, data security and patient privacy continue to challenge its widespread adoption.

This review explores the historical context, current applications, and future directions of telecardiology, examining its transformative effects on cardiovascular medicine. Through a detailed analysis of the technological advancements and clinical applications, this study aims to elucidate the benefits of telecardiology and provide insights into the potential challenges of integrating telecardiology into mainstream cardiovascular care, providing insights for healthcare providers, policymakers, and researchers in the field.

## Review

Telecardiology is gradually emerging as a significant component of modern cardiovascular care, offering numerous benefits by integrating telemedicine into cardiology practice. One of the significant benefits of telecardiology is the improved access to healthcare services and the convenience it provides, particularly for patients in geographically isolated or rural areas or those that are physically limited, such as those with disabilities or even communicable infections [8]. By eliminating the need for extensive travel, telecardiology significantly reduces travel time and associated costs, making it easier for patients to attend appointments [9] and improving health-seeking behavior and health outcomes. Also, the flexibility of telemedicine appointments, which can be scheduled during evenings or weekends, accommodates patients' busy schedules, further enhancing accessibility. This is especially beneficial for those who might otherwise forgo regular check-ups due to logistical challenges, ensuring continuous and consistent care [10].

In addition to the convenience it brings, telecardiology significantly enhances patient management. Through telecardiology, clinicians can now continuously track vital signs and detect early signs of complications, allowing for timely interventions through remote monitoring technologies, such as electrocardiograms (ECGs) and blood pressure monitors. Through real-life data sharing with clinicians, patients can be notified about warning signs and abnormal rhythm patterns, providing a timely expert intervention before potentially fatal outcomes, thus improving global health indices. Technological advancement through wearable technology and mobile health applications is critical to allow real-time feedback and foster a more engaged and informed patient population. This proactive approach is crucial in managing chronic cardiovascular conditions, improving medication adherence, and educating patients on self-management practices [11]. Therefore, as expected, there are notable positive clinical outcomes associated with telecardiology, including reduced hospital readmissions, improved quality of life, and increased patient satisfaction through early detection of heart conditions, health-seeking behavior, and early intervention. Early intervention capabilities for conditions like heart failure and arrhythmias mean that potential issues are addressed before they escalate into more severe problems [12]. This also, in turn, proves to be cost-effective as it reduces healthcare expenditures through fewer hospital stays and more efficient use of specialist resources [13]. Telecardiology is a vital part of preventive medicine. It promotes significant long-term savings by limiting costly medical procedures and the cost of seeking healthcare services, such as transportation, missed work schedules, and emergency room visits.

Telecardiology is, however, not without potential challenges. The risk of worsening health disparity continues to be a significant concern, especially among low-income populations, due to the cost of implementation, technological infrastructures that support telemedicine, and health literacy needed for operation and awareness. Also, as discussed previously, concerns regarding patients' sensitive data and privacy concerns pose significant barriers to its adoption. Integrating telecardiology with traditional in-person care models is essential to create a cohesive and comprehensive approach to cardiovascular health management.

Technological innovations in telecardiology

The telecardiology world continues to evolve with technological advancements. We have seen significant technological innovations, particularly in remote monitoring devices. Advances in telecommunication, including improved bandwidth and the internet, have facilitated high-quality video consultations, real-time data transmission and analysis, and robust security protocols [11]. This has further enhanced precise and reliable video conferencing, making real-time consultations and secure data transmission more reliable. These advancements enable cardiologists to conduct thorough virtual examinations and consultations, which are equivalent to in-person visits, thus expanding access to specialized care and timely interventions [11]. A section of telecardiology that is recently gaining more attention is the telerehabilitation platform that provides a structured, remote cardiac rehabilitation program including exercise guidance and progress monitoring, especially in patients with significant cardiac events such as heart attacks (myocardial infarction), heart failure, or debilitation arrhythmias [14]. This has helped with the recuperation process as well as secondary prevention. The telerehabilitation platform enhances patient engagement and rehabilitation due to its convenience and fewer logistic concerns that may hinder in-person participation. This has improved the uptake of cardio-rehabilitation programs and potentially better health outcomes [14].

Additionally, emerging technologies like virtual reality for immersive cardiac rehabilitation exercises and the development of implantable monitoring devices hold a promising future for the world of telecardiology through potential remote surgery and more personalized preventive care, which will be a reality in the near future. These innovations reinforce advanced technology's transformative impact on cardiovascular care, which improves outcomes and expands access to life-saving interventions. Using telemedicine and mobile services helps bridge the geographical gap between patients and healthcare providers as well as health-seeking behavior, which is paramount to improving health outcomes [15].

Concurrently, AI has also emerged as a pivotal component in telecardiology, enhancing diagnostic imaging, particularly in ECG data analysis and abnormality detection. This is critical for early diagnosis and intervention of cardiac pathologies. AI algorithms can rapidly process vast amounts of cardiovascular data, including the identification of "warning" patterns that might indicate potentially fatal conditions such as atrial fibrillation or myocardial infarctions with significant accuracy [16]. This ability has helped improve diagnostic precision and enables timely interventions, which provides critical prognostic value for managing acute cardiac events and chronic conditions. Moreover, AI-driven predictive analytics can forecast potential health issues, enabling preemptive measures that could potentially improve patient outcomes and, in turn, reduce healthcare costs [16].

Furthermore, integrating mobile health (mHealth) technologies has further expanded the scope of telecardiology. Smartphone applications now play a crucial role in data collection, medication adherence, and patient education, empowering patients to participate in their care actively. These mobile apps can monitor vital signs, serve as reminders for medications, thus improving medication compliance, and offer educational resources about managing heart conditions. By fostering a collaborative approach to healthcare, mHealth technologies help patients and providers work together more effectively to manage cardiovascular health.

Wearable cardiotechnology is another potential game changer in the telecardiology field through the advent of devices such as smartwatches and fitness trackers, which use in-built sensors that can monitor heart rate and activity levels and even detect irregular heart rhythms such as atrial fibrillation, potentially improving early health-seeking behavior, intervention, and favorable outcomes [1]. These wearable technological devices can also provide continuous health data that can be shared with healthcare providers in real-time, allowing for more responsive and personalized care. Of note, some of these devices can store information that can be shared with providers during visits, aiding in diagnosing and treating medical conditions. The continuous monitoring capability is particularly beneficial for patients with chronic cardiovascular conditions as it allows early detection of potential issues and timely interventions.

In addition to the above-mentioned innovations, through cloud computing improvements, healthcare providers can now access and analyze stored large datasets, which can be used to track patient outcomes over time, identify trends, and formulate personalized treatment plans that can be potentially helpful for improved health outcomes. This also facilitates better communication and data sharing among providers, improving a coordinated and collaborative healthcare approach. Table 1 summarizes the technological innovations in telecardiology.

**Table 1:** Technological innovations in telecardiology Source: This table was created by Francis Ezekwueme, one of the authors.

Technology	Description	Advantages in telecardiology	Potential limitations
Remote patient monitoring (RPM)	Involves the transmission of important patient health data, including vitals and weights, with providers. Some of the FDA-approved RPMs are the implanted pacemakers and defibrillators.	This promotes continuous patient monitoring, early disease detection and intervention, and reducing hospital admissions.	Health literacy/patient education, cost of compatible devices, data security, and privacy measures
Teleconsultation	It includes video or audio consultations between patients and cardiologists.	This improves access to specialists, improves convenience by reducing travel burden, and facilitates chronic disease management.	Bandwidth and internal access and risk of impersonal interaction
Mobile Health (mHealth)	It involves using mobile devices such as smartphones and tablets for health apps and education. KardiaMobile Device is currently FDA-cleared.	It is easy to use and access and improves patient engagement, self-care, and medication adherence through reminders.	Concerns for data privacy, app security, digital/health literacy gap, and cost are potential barriers.
Wearable devices	Smartwatches, such as the Apple Watch, are fitness trackers that monitor heart rate and activity levels. Some of the FDA-cleared devices are the Apple Watch series 4-8, Google Pixel Watch, Samsung Galaxy Watch 3, and Samsung Galaxy Watch Active 2.	It can provide real-time health data and facilitate the early detection of arrhythmias, abnormal rhythms and early health-seeking behavior.	Cost is a significant barrier, especially for low-income earners. Battery life, accuracy concerns, and data overload.
Telerehabilitation	It involves cardiac rehabilitation programs, which are delivered virtually.	It improves access to rehabilitation services as well as convenience by reducing travel burden and other logistics.	Limited physical interaction. Requires home equipment, which improves compliance and patient motivation.
Artificial intelligence (AI) in telecardiology	It involves AI algorithms for ECG analysis and risk prediction.	It augments decision-making and streamlines workflows and personalized care.	Transparency and explainability of AI models, the potential for bias, privacy, and health data security are potential barriers.

Telecardiology in chronic cardiovascular disease management

Heart Failure Management

One of the most significant benefits of telecardiology is its potential to manage chronic cardiovascular diseases, particularly heart failure [17]. It allows for remote monitoring systems that enable real-time tracking of patients' vital signs, such as weight, blood pressure, and heart rate, which are crucial for managing heart failure. These findings alert healthcare providers to early signs of decompensation, allowing timely interventions to prevent hospitalizations and improve patient outcomes [18]. Studies have shown that telemonitoring can reduce heart failure-related hospital admissions and mortality rates through early decompensation detection and proactive management of the condition [19]. In addition, integrating these systems with patient education programs enhances self-awareness and management skills, allowing patients to take an active role in their care. This provides a holistic approach that helps improve clinical outcomes and enhances the quality of life for patients with heart failure.

Arrhythmia Management

Detecting and monitoring arrhythmias, including atrial fibrillation, is another area where telemedicine plays a crucial role. Remote monitoring devices, such as wearable ECG monitors or other wearable health devices, allow for continuous surveillance of cardiac rhythms [18]. These telecardiology devices promote the early detection of arrhythmic events as well as prompt medical response, thereby reducing the risk of potentially fatal health outcomes such as stroke and other complications [18]. Additionally, telecardiology facilitates regular follow-ups and medication adjustments, ensuring better control of arrhythmias and enhancing patient safety through its convenience in scheduling visits and avoiding logistics hindering in-person visits. Advanced algorithms in these devices can analyze rhythm data in real-time, providing actionable insights for patients and healthcare providers. This continuous feedback loop is critical for maintaining optimal heart rhythm control and reducing potentially fatal adverse events associated with arrhythmias [18].

Hypertension Management

Hypertension remains one of the most critical risks for major cardiovascular diseases. Hypertension can be effectively managed through telemedicine through home blood pressure monitoring devices connected to telehealth platforms, thus providing accurate, real-time, and frequent blood pressure that can help create personalized treatment plans, which should improve health outcomes [20]. Continuous blood pressure monitoring allows healthcare providers to make data-driven decisions regarding antihypertensive therapy adjustments based on trends from readings. This has also led to improved adherence to treatment plans and blood pressure control, which in turn leads to a reduced risk of hypertension-related cardiovascular complications such as heart failure, stroke, or even myocardial infarction [21]. In addition, these platforms also provide health education on dietary and lifestyle modifications, which are crucial for managing hypertension. Integrating behavioral coaching with telemonitoring has been shown to improve patient engagement and long-term hypertension control and management significantly.

Cholesterol Management

Another significant risk factor for cardiovascular complications is elevated cholesterol levels (dyslipidemia), which can be closely managed through telemedicine. Through virtual consultations, healthcare providers can offer personalized dietary and lifestyle recommendations, prescribe lipid-lowering medications, and monitor patients' lipid profiles. These processes are crucial for maintaining a safe lipid profile and are critical to reducing dyslipidemia-related cardiovascular complications [22]. Studies have also backed the benefits of telecardiology in managing dyslipidemia, further reinforcing its benefit in management [22]. The convenience of remote lipid monitoring and follow-up has also improved adherence to recommendations and reduced the risk of missed appointments by scheduling and attending in-person visits. Educational programs can also be provided remotely, thus maintaining optimal cholesterol levels and preventing complications that arise from poorly controlled lipid levels.

Medication Adherence

Continued medication reconciliation and adherence remain critical to chronic cardiovascular disease management. Telemedicine provides tools such as electronic reminders, virtual follow-ups, and remote medication management, which enhance adherence to prescribed therapies [23]. Studies have shown better clinical improvement, improved medication adherence, reduced hospital admissions and readmissions, and lower mortality rates [24]. Telemedicine platforms can offer personalized adherence support through automated messages and direct communication with healthcare providers. These also help improve medication refills, preventing patients from running out of their medications and improving medication compliance and health outcomes. Telemedicine enhances patient adherence and overall disease management by providing real-time support and accountability [24].

Comprehensive Care Integration

The integration of telemedicine into cardiovascular care models has the potential to transform the management of chronic cardiovascular conditions [20]. By combining remote monitoring, virtual consultations, patient education, and adherence support, telemedicine creates a comprehensive care network beyond traditional in-person visits. This holistic approach ensures continuous and proactive management of cardiovascular health, improving patient outcomes and reducing the burden on healthcare systems. As telemedicine technologies continue to advance, their role in cardiovascular care will likely expand, offering new opportunities for innovation in disease management and patient engagement [20].

Teleconsultations and virtual follow-ups

Secure Video Conferencing Platforms

Secure video conferencing platforms are central to telemedicine in cardiology, enabling real-time, face-to-face interactions between patients and healthcare providers. They are essential for patients with chronic cardiovascular diseases such as hypertension, heart failure, and arrhythmias, allowing for regular surveillance and monitoring and providing real-time interventions as needed [9]. These platforms ensure comprehensive assessment and management of cardiovascular conditions through high-quality audio and video capabilities. They are also designed to comply with data privacy regulations, such as the Health Insurance Portability and Accountability Act (HIPAA), in the United States, ensuring secure and confidential communication [25]. This compliance is critical for maintaining patient trust and protecting sensitive health information. Moreover, these platforms often include features like screen sharing and digital stethoscope integration, which enhance the virtual examination process, allowing for remote, thorough, and accurate clinical assessments. Regular follow-up promotes management compliance and overall improved health outcomes.

Virtual Care Delivery Models

Virtual care delivery models in cardiology encompass both synchronous (live video consultations) and asynchronous (store-and-forward) telemedicine services [26]. Synchronous consultations allow immediate interaction and real-time decision-making, which is crucial in urgent and complex cases. Asynchronous telemedicine services, on the other hand, enable patients to send their medical data, such as ECG recordings or blood pressure logs, to healthcare providers for later review, providing flexibility and convenience. These models offer flexibility and accessibility, allowing patients to receive care from the comfort of their homes. They are particularly beneficial for managing chronic conditions, enabling continuous monitoring and timely interventions [27]. Virtual care delivery models also reduce the burden on healthcare facilities, improve patient access to specialists, and enhance the overall efficiency of the healthcare system. A potential downside of the asynchronous telemedicine service is a delay in intervention compared to the synchronous telemedicine services.

Patient Portals

Patient portals are another integral part of telemedicine, providing patients access to their health care records, lab results, imaging reports, and treatment plans. It also offers the benefit of communicating with healthcare providers for concerns regarding their health, medication refill requests, receiving educational materials tailored to their health conditions, and scheduling appointments. This also tends to improve physician-to-patient relationships while providing an avenue for timely interventions as needed. In addition, the accessibility to their health care also allows patients to make informed decisions regarding their health and active participation in their health care.

E-Prescribing Medications

E-prescribing through telemedicine platforms simplifies the process of prescribing and managing medications. It allows healthcare providers to send prescriptions electronically to pharmacies, thereby reducing errors and improving efficiency. This feature supports remote medication management and adherence by providing a seamless and streamlined medication order and refill process. E-prescribing systems often include decision support tools that alert providers to potential drug interactions and allergies, enhancing patient safety. Additionally, patients benefit from the convenience of having their prescriptions ready for pickup or delivery without the need for physical paper prescriptions. On the patient's part, this reduced the risk of running out of medications and improved medication and management compliance, potentially improving health outcomes.

Remote Medication Management

Remote medication management involves monitoring patients' medication use and adherence through telemedicine platforms. Healthcare providers can track prescription refills, assess side effects, and make necessary adjustments to treatment plans. This approach enhances medication adherence and ensures optimal therapeutic outcomes. By using telemedicine tools, providers can set up automated reminders for patients to take their medications and follow up on their adherence through virtual consultations. Remote monitoring can detect non-adherence early and allow for interventions to address barriers, such as forgetfulness or side effects, thereby improving the effectiveness of treatment regimens.

Patient Engagement and Continuity of Care

Telemedicine fosters patient engagement by providing accessible and convenient care options. Continuous communication and regular follow-ups through telemedicine platforms ensure patients remain engaged in their treatment plans. This continuity of care improves clinical outcomes and enhances patient satisfaction. Features like video visits, secure messaging, and access to personalized health information keep patients connected with their healthcare teams. Engaged patients are more likely to adhere to treatment plans, attend follow-up appointments, and maintain healthy behaviors. Telemedicine also offers educational resources and support groups, which can help patients manage their conditions more effectively and feel more supported in their healthcare journey.

Patient satisfaction and outcomes

Improved Adherence to Treatment Plans

Telemedicine interventions have improved adherence to treatment plans [28]. Remote monitoring and virtual follow-ups ensure that patients receive consistent care and support, leading to better adherence and improved clinical outcomes. These interventions include but are not limited to automated reminders, personalized health coaching, and regular check-ins via telehealth platforms, all of which help patients stay on track with their prescribed therapies. Facilitating more accessible communication between patients and providers can improve physician-patient trust while addressing medication side effects or misunderstandings about treatment regimens, which can otherwise hinder adherence [28].

Data on Clinical Outcomes

Studies have shown that telemedicine can positively impact clinical outcomes in cardiology through multifaceted means [17]. For instance, remote monitoring of heart failure patients has been associated with reduced mortality rates through patient monitoring and early detection of warning signs and treatment as needed [29]. These findings highlight the effectiveness of telemedicine in improving patient outcomes. Studies have also shown that telemedicine can lead to better blood pressure control, improved lipid levels, and reduced incidence of cardiovascular events, as discussed above. This is achieved through continuous monitoring, timely adjustments to treatment plans, and immediate medical advice when needed, collectively contributing to better disease management and outcomes.

Quality of Life

Telecardiology enhances the quality of life for cardiovascular patients by providing convenient and timely access to care. Patients can manage their conditions more effectively, experience fewer complications, and enjoy a better overall quality of life. With telemedicine, patients can avoid the stress and inconvenience of frequent travel to healthcare facilities, which is particularly beneficial for those with mobility issues or living in remote areas. Additionally, the flexibility of telehealth appointments allows patients to integrate their healthcare more seamlessly into their daily lives, reducing the disruption caused by medical visits and promoting follow-up compliance and management adherence, which have been shown to improve health outcomes.

Reduced Hospital Readmission Rates

Telecardiology has been linked to reduced hospital readmission rates for cardiovascular patients [17] through continuous monitoring and timely interventions to help prevent disease exacerbations, hence reducing the need for hospital readmissions. For example, remote monitoring can detect early signs of heart failure decompensation, prompting preemptive adjustments in treatment, such as increases in the dose of diuretics in a patient with increasing weight/fluid retention noted through continued monitoring, which are subtle signs of decompensated heart failure, averting hospital admissions. This enhances patient outcomes and reduces the strain on healthcare systems, freeing up resources for other critical needs.

Increased Patient Engagement

Telemedicine promotes increased patient engagement by offering accessible patient-centered care [30]. Engaged patients are more likely to adhere to treatment plans, participate in their care, and achieve better health outcomes. Telehealth platforms often include features such as educational resources, interactive health tools, and direct communication channels with healthcare providers. These features further empower patients to take active health management roles, fostering a sense of responsibility and ownership over their care. Increased engagement also leads to better patient-provider relationships, crucial for effective long-term disease management.

Cost-Effectiveness Analysis

A cost-effectiveness analysis of telemedicine in cardiology demonstrates its financial benefits. By reducing hospital readmissions, travel costs, and time off work, telemedicine can offer significant cost savings for both patients and healthcare systems. Table 2 summarizes the cost-effectiveness of telemedicine interventions in cardiology [31,32]. Telemedicine reduces the need for in-person visits and the associated costs, such as transportation and lost productivity (labor productivity due to time off from work during an emergency room visit or hospitalization). Furthermore, telemedicine contributes to overall healthcare cost savings by improving disease management and reducing acute episodes requiring expensive emergency care. Telemedicine can optimize resource allocation for healthcare systems, allowing for better healthcare personnel and facility management.

**Table 2:** Cost-effectiveness of telemedicine interventions in cardiology Source: This table was created by Francis Ezekwueme, one of the authors.

Parameters	Telemedicine	Traditional care
Hospital readmission rates	Reduced	Higher
Travel costs	Reduced	Higher
Time off work	Reduced	Higher
Overall healthcare costs	Reduced	Higher

Additional considerations

Rural Healthcare Disparities

The primary adoption of telemedicine was to provide healthcare services for those in remote and underserved regions. Telecardiology addresses rural healthcare disparities by providing access to specialized cardiology care for patients in remote areas. This technology bridges the gap between rural and urban healthcare systems, ensuring patients in geographically isolated locations receive the timely and appropriate care they deserve [33]. Through telemedicine, rural patients can consult with cardiologists and other specialists without extensive travel, which is often a significant barrier to healthcare access. This is particularly important for managing patients with chronic conditions, where consistent monitoring and follow-up are critical. Implementing telecardiology in rural areas also allows local healthcare providers to collaborate with specialists, enhancing the overall quality of care delivered and thus promoting a collaborative and holistic approach to care.

Health Equity

Telemedicine promotes health equity by offering accessible, affordable care options for underserved populations. It has been shown to reduce barriers to care, such as transportation, time constraints, and bodily and financial limitations, ensuring all patients have equal opportunities to receive high-quality cardiovascular care [34]. By leveraging telehealth technologies, healthcare providers can reach vulnerable populations with limited access to medical services due to socioeconomic factors. This approach improves individual health outcomes and contributes to broader public health goals by addressing systemic inequities in healthcare access and delivery, which is one of the critical objectives of Healthy People 2030.

Challenges and barriers to implementation

Technological Limitations and Infrastructure Requirements

Telemedicine significantly improves the healthcare system, particularly in cardiology, through improved access to preventive treatment, thereby enhancing long-term health outcomes. It facilitates medical attention at the convenience of patients and doctors, reducing the need for logistical arrangements such as taking time off work or arranging childcare to attend appointments. Additionally, telemedicine minimizes exposure to infectious agents commonly encountered in hospital settings [35].

One study highlighted challenges such as insufficient bandwidth, poor telecommunications infrastructure, and inadequate access to necessary materials like computers and software [36]. These issues, alongside misaligned organizational goals and user expectations, pose significant barriers to effective telemedicine implementation [36]. Similarly, a study conducted in Japan identified weak health information infrastructures, difficulty in evaluating system cost-effectiveness, the imperfect nature of human-machine communication, low internal speed, poor audio or video streaming quality, and suboptimal conditions of equipment and medical devices as significant limitations [37]. Another study echoed these concerns, emphasizing bandwidth issues, weak telecommunications, and the lack of available clinical devices as critical obstacles [38].

In the field of cardiology, these limitations can potentially hinder the effective management of cardiovascular diseases, where timely and accurate data transmission is critical to prognostic value. Addressing these challenges involves a multifaceted approach, which includes improving telecommunications infrastructures, investing in robust health information systems, and ensuring access to high-quality clinical devices [38]. These efforts are essential to fully realize the optimal benefits of telemedicine in enhancing cardiac care and improving patient outcomes.

Regulatory and Reimbursement Issues

Reimbursement remains a significant barrier to telemedicine, particularly in cardiology. For instance, Medicare reimbursement is restricted for telehealth visits due to concerns over potential overutilization and abuse of the healthcare system [39]. This limitation poses significant challenges for cardiologists who wish to utilize telemedicine for routine follow-ups and management of chronic cardiovascular conditions. Additionally, many technological companies are entering the telemedicine market primarily for financial gain rather than to enhance healthcare delivery, which may affect the quality and focus of telemedicine solutions available to cardiologists [39].

Licensing also remains a critical barrier in telemedicine. Many states require an in-person consultation before any subsequent telehealth visits, which aligns with the American Medical Association's (AMA) guidelines [39]. This restriction complicates providing continuous and seamless cardiology care, particularly for patients in rural or underserved areas who may benefit most from telehealth services. Furthermore, telemedicine frequently encounters regulatory barriers from traditional agencies such as the Food and Drug Administration (FDA), which can delay the approval and deployment of innovative telehealth technologies and devices critical for cardiology [39].

Additionally, the Social Security Act limits the use of telemedicine to specific providers, further restricting access to telehealth services for patients requiring cardiology care [39]. These regulatory and reimbursement challenges hinder telemedicine's widespread adoption and integration in cardiology despite its potential to significantly improve patient outcomes through enhanced monitoring, timely interventions, and better management of cardiovascular diseases.

Addressing these barriers requires comprehensive policy reforms. Expanding Medicare and other insurance reimbursements for telehealth visits, especially for chronic disease management in cardiology, is essential to maintain the global adoption of telemedicine in cardiology. Additionally, simplifying and standardizing licensing requirements across states can facilitate broader access to telehealth services, especially for those in rural and underserved populations. Also, updating regulatory frameworks and policies to keep pace with technological advancements can ensure cardiovascular disease patients benefit from the latest telemedicine innovations. Such reforms are crucial for overcoming the current obstacles and fully leveraging telemedicine to improve cardiovascular health outcomes [39].

Ethical Considerations in Telecardiology

Ethical considerations in telecardiology are multifaceted and have gained increased importance as the adoption of telemedicine increases. Data privacy and security are paramount, particularly with increased cyber threats and HIPAA regulations [40]. Healthcare providers must employ robust encryption and secure communication platforms to protect patient information, maintain patient trust, and comply with legal requirements [40]. Additionally, securing telehealth platforms against unauthorized access and breaches is crucial for safeguarding patient data.

It is also critical to understand that obtaining informed consent in telecardiology involves the usual consent for treatment and explicit consent for using digital communication tools, which may be used for remote monitoring and treatment. Patients must be informed about the risks and benefits of telehealth, including potential issues related to data security and privacy [41]. This ensures that patients can make well-informed decisions about their care and decide to pull out at their discretion.

Not only that, there is also a growing focus on equity and access, ensuring that telecardiology services are available to all populations, including those in underserved and rural areas, thus promoting fairness and reducing healthcare disparities. Telemedicine should be designed and implemented so as not to exacerbate existing inequities. For instance, efforts should be made to provide the necessary technology and internet access to underserved communities and to create user-friendly telehealth platforms that accommodate patients with varying levels of digital literacy. This is, however, challenging as many rural or underserved populations still do not have the necessary resources to optimize telecardiology due to a lack of funding, good internet access, excellent bandwidth for video and audio consultations, and digital literacy.

Broader Ethical Considerations

Beyond privacy and access, ethical considerations in telecardiology also include ensuring the quality and continuity of care. Providers must be diligent in maintaining high standards of care in virtual settings, ensuring that telehealth visits are as thorough and effective as in-person consultations [35]. This includes using advanced diagnostic tools and ensuring proper follow-up care.

Another ethical issue is the potential for telemedicine to inadvertently reduce the patient-provider relationship's personal touch, which is crucial in cardiology, where ongoing patient engagement and trust are vital. Strategies to mitigate this include using video conferencing to maintain face-to-face interactions and ensuring that telehealth complements rather than replaces necessary in-person visits. This is, however, less likely in areas with lesser technological advancements, further bridging health disparities.

Furthermore, ethical telecardiology practice involves ongoing education and training for healthcare providers to stay updated with technological advancements and best practices in telehealth. This ensures that providers can deliver the highest quality care and navigate the unique challenges of remote patient management.

Successful telecardiology programs and initiatives

The adoption of telemedicine gained significant recognition since the coronavirus disease 2019 (COVID-19) pandemic due to a saturated healthcare system at that time as its use became prominent through global efforts to reduce the spread of the fatal virus through isolation protocols. However, its roots extend back much further. Telemedicine was instrumental in managing major historic epidemic outbreaks, such as the severe acute respiratory syndrome (SARS) epidemic in Taiwan, the H1N1 and H7N9 influenza pandemics in China, and the Middle East respiratory syndrome (MERS) outbreaks [42,43]. Similar to the adoption during the COVID-19 pandemic, it was created as an alternate means for disease surveillance, patient monitoring, and follow-up while still curbing the spread of the disease. Through telemedicine, patients can still access the healthcare system remotely and safely without exposing themselves to the fatal virus.

In Sub-Saharan Africa (SSA), telemedicine programs have historically supported and strengthened healthcare delivery, patient and professional health education, and disease prevention and surveillance [42,43]. For example, similar to other pandemics, telemedicine played a critical role during the Ebola virus outbreak by helping to limit the spread of the disease while providing a platform for surveillance and emergency treatment. The first adoption of telemedicine in SSA occurred in Ethiopia in 1980 under the HealthNet project [42].

In the United States, telemedicine continues to have a robust impact in the cardiology field, partly due to the prevalence of cardiovascular disease. At-home monitoring programs for cardiology patients have proven highly effective, especially for those with chronic cardiac conditions such as heart failure or abnormal heart rhythms [18]. These programs facilitate video conferencing, real-time data acquisition, and sharing with physicians for interpretation and management [44]. Telemedicine devices such as Holter monitors, blood pressure monitors, and implantable cardioverter-defibrillators have significantly reduced mortality rates. Over six years, some home telemedicine monitoring programs have reduced hospital readmissions by 44% and saved over $10 million [44,45]. These programs also enhance healthcare efficiency by allowing medical personnel to attend to more patients concurrently [44].

During the COVID-19 pandemic, many hospitals and clinics transitioned to telemedicine to mitigate the spread of the virus through isolation protocols. A study at New York University (NYU) Langone Health in the United States reported an 80% decrease in in-person visits and a 683% increase in telemedicine visits between March 2, 2020, and April 14, 2020 [46]. Electronic intensive care units (e-ICUs), which were created, enabled clinicians to monitor and manage large patient loads simultaneously as demonstrated by Aurora Health in Wisconsin [46]. Mobile home healthcare units are also being developed to safely evaluate and manage patients who can return home, reducing emergency room saturation and overcrowding [44].

In cardiology, telemedicine has revolutionized the management of chronic cardiovascular conditions through remote patient monitoring and real-time management. Patients with heart failure, arrhythmias, and hypertension benefit from remote monitoring and real-time data transmission to their healthcare providers. This continuous monitoring allows for timely interventions, reducing the risk of potentially fatal adverse events. For instance, implantable cardiac devices can transmit data wirelessly to healthcare providers, which enables the early detection of arrhythmias and other cardiac events, leading to prompt treatment adjustments and improved patient outcomes [44].

In addition, telemedicine facilitates cardiac rehabilitation, an essential component of care for patients recovering from cardiac events [14]. Telecardiology has provided patients with personalized exercise plans, dietary recommendations, and regular virtual consultations with healthcare providers through remote cardiac rehabilitation programs [14]. This approach has helped improve adherence to rehabilitation protocols and enhance recovery, especially for remote, isolated, or underserved patients.

Telemedicine has also proven valuable in rural and underserved regions with limited access to specialized cardiology care. Remote consultations and diagnostics enable cardiologists to extend their expertise to patients who otherwise face significant barriers to care [44]. This increased accessibility has the potential to reduce disparities in cardiovascular health outcomes.

Future directions

Through advanced technologies such as AI and machine learning, greater integration of these technologies into the world of cardiology is expected. This will enhance diagnostic accuracy and personalized treatment plans based on real-time data analysis and predict patient outcomes, which will be critical for improving the overall efficiency of the healthcare system. Also, advances in wearable technology and implantable devices will help expand remote monitoring capabilities, which will benefit cardiology patients with physical limitations such as transportation issues, frailty, and work-life issues, thus improving access to healthcare in these populations. These also reduced the wait time on clinic/emergency room visits, delays in seeking healthcare, and overcrowding in healthcare facilities. With continued research, future devices are expected to provide more comprehensive and continuous data on various cardiovascular parameters, enabling more proactive and preventive care.

With the security and privacy of patient data being a significant concern to telemedicine, future telemedicine systems will need to incorporate robust security measures to protect sensitive health information while still providing the propelling benefits of telemedicine. Although there are ongoing measures for cybersecurity, with continued research, this is expected to improve. Although not well established at this time, the development of telemedicine protocols for emergency cardiology care, such as remote consultation during acute cardiac events, could potentially improve patient outcomes through early, more accessible care with experts providing a timely intervention in critical situations, hence improving the overall health outcome of cardiology emergencies. Building global telemedicine networks is also paramount to allow for international collaborations and knowledge dissemination, which in turn help standardize protocols and provide access to specialized expertise worldwide.

Although detailed examination practices, reimbursement concerns, and telemedicine overuse continue to hinder its acceptance, continued evolution will require supportive policies, reimbursement policies, and regulatory changes to help fasten its integration into mainstream healthcare. This also, in turn, promotes the generalized uptake of telemedicine in cardiology. Another significant barrier to the widespread adoption of telemedicine is the lack of awareness of its benefits. Future directions should aim to provide ongoing education and training programs for patients and healthcare providers, educating them on the benefits of telemedicine and the process of integrating telemedicine into their clinical practices. Providing the necessary support needed to make this integration as easy as possible could, in turn, increase its overall adoption.

Research on the efficacy and outcomes of telemedicine in cardiology is limited at this time, and efforts should be directed at building robust evidence-based studies that support the optimization and standardization of telemedicine practices and ensuring that they meet the highest standards of care. With continued research, it is undeniable that the robust benefits of healthcare could result from the global adoption and integration of telemedicine in cardiology.

## Conclusions

Telemedicine is becoming integral to cardiology, enhancing patient management, monitoring, and overall healthcare efficiency. Although pivotal in managing epidemic outbreaks, telemedicine has become vital for managing and monitoring chronic disease management, particularly in cardiology. It has facilitated remote monitoring, reduced hospital readmissions, and significantly improved healthcare. The COVID-19 pandemic was key to the adoption of telecardiology because it could manage large patient loads and maintain care continuity during crises. Technological advancements, data security, and global collaboration will further solidify telemedicine's role in cardiology, ensuring more accessible, efficient, and personalized patient care in the United States and worldwide.
